# Flow and access: Driving forces of COVID-19 spreading in the first stage around Hubei, China

**DOI:** 10.1371/journal.pone.0280323

**Published:** 2023-01-20

**Authors:** Tianhai Zhang, Jinqiu Cao

**Affiliations:** 1 Engineering College, Sichuan Normal University, Chengdu, China; 2 West China School of Nursing, Sichuan University, Chengdu, China; Southwest Jiaotong University, CHINA

## Abstract

**Background:**

This research takes the six provinces around Hubei Province where the Corona virus disease 2019 (COVID-19) outbreak as the research area, collected the number of cumulative confirmed cases (NCCC) in the first four weeks after the lockdown to explore the spatiotemporal characteristics, and to identify its influencing factors by correlation and regression analysis, finally providing reference for epidemic prevention and control policy.

**Methods:**

The analysis of variance was used to test the spatiotemporal variability of the NCCC in the six provinces, the Pearson coefficient was taken to find the correlation relationship between the NCCC and multiple factor data in socio-economic, geography and transportation, and the following regression equation was obtained based on regression analysis.

**Results:**

**T**his study found that there is significant spatial variability in the NCCC among the six provinces and the significant influencing factors are changing along the four weeks. The NCCC in Shaanxi and Chongqing in the West was less than that in the other four provinces, especially in Shaanxi in the northwest, which was significantly different from the four provinces in the East, and has the largest difference with adjacent Henan province (792 cases). Correlation analysis shows that the correlation coefficient of the number of main pass is the largest in the first week, the correlation coefficient of the length of road networks is the largest in the second week, and the NCCC in the third and fourth week is significantly correlated with the average elevation. For all four weeks, the highest correlation coefficient belongs to the average elevation in the third week (r = 0.943, P = 0.005). Regression analysis shows that there is a multiple linear regression relationship between the average elevation, the number of main pass and the NCCC in the first week, there is no multiple linear regression relationship in the second week. The following univariate regression analysis shows that the regression equations of various factors are different. And, there is a multiple linear regression relationship between the average elevation, the length of road networks and the NCCC in the third and fourth week, as well as a multiple linear regression relationship between the average elevation, population and the confirmed cases in the fourth week.

**Conclusion:**

**T**here are significant spatial differences in the NCCC among the six provinces and the influencing factors varied in different weeks. The average elevation, population, the number of main pass and the length of road networks are significantly correlated with the NCCC. The average elevation, as a geographical variable, affects the two traffic factors: the number of main pass and the length of road networks. Therefore, the NCCC is mainly related to the factor categories of flow and access.

## Introduction

Since the outbreak of Corona virus disease 2019 (COVID-19), global public health and safety are facing great challenges [1–3]. As of July 4, 2022, the number of cumulative confirmed cases (NCCC) exceeded 500 million, and cumulative deaths exceeded 6 million cases. Therefore, detecting the diffusion pattern of this epidemic, identifying its influencing factors and then making according risk assessments to prevent or control the ongoing COVID-19 is a key issue not only to this COVID-19 but also to future similar epidemic.

In the early days after the outbreak of COVID-19 epidemic, many scholars focused on biology and virology [4–6], or epidemic monitoring and risk management [7–9], which contributed greatly to the COVID-19 prevention. Most of the modeling prediction focused on Wuhan, China [10, 11], but there’s also some research about Europe and Americas [12–14].

For better coping with the COVID-19 epidemic and risk assessments, in addition to model simulation and prediction, it is also very important to understand the spatiotemporal pattern of COVID-19 in the early stage and its influencing factors [15]. On identification the spatiotemporal distribution characteristics and changing trends of the epidemic, some scholars detected the spatial aggregation with autocorrelation or described the statistical analysis [16–18]. The research scales include whole word, countries, provinces, cities, districts and communities [19–22]. Some of These studies show that the spatial aggregation of epidemic distribution has different characteristics at different scales, which is worthy of further study [16, 19].

Based on the detection of spatial distribution, the influencing factors can be further identified. The main idea is to correlate the case information with various factors to detect which factors affected the transmission of the epidemic. These factors generally include climate factors, environment factors and socio-economic factors [22, 23]. On climate and environment factors, research conclusion in different regions and scales are quite different [22]. Wang found that the daily average temperature is negatively correlated with COVID-19 and the air pollution were positively correlated with COVID-19 in China [17]. Coccia also point out that air pollution has a high association with diffusion of COVID-19 in North Italy [23]. But Jahangiri concluded that temperature have low sensibility with COVID-19 in Iran [24]. Rahman also revealed that at the global scale the temperature was not related with the spatial variability of COVID-19 spread [22].

For socio-economic factors, many studies have pointed out that population have affected the spread of COVID-19 [15, 19, 24, 25]. Jia and Xie found the population flow were the main factors affecting the epidemic spread [15, 18]. But Jahangiri, Sannigrahi and Xiong concluded that total population is significant factor association with COVID-19 [19, 24, 25]. Xiong also found that at the prefecture level in Hubei province, the COVID-19 has no association with population density but at the county level the COVID-19 had a positive and extremely significant correlation (p < 0.01) with population density. In the European region, Sannigrahi suggests that poverty and income are the key factors in regulating COVID-19 [25].

In the research on the spatial characteristics and influencing factors of COVID-19 epidemic, current studies pay more attention to the spatial aggregation, but less attention to the spatiotemporal variability between adjacent regions; Most of research are based on national, prefecture and county level, few studies focus on neighboring provinces in the early outbreak stage. In addition, few researches set up comprehensive variables system integrate multiple dimensions of social-economy, geography and transportation [18, 19, 26, 27]. Further, this research has a hypothesis that the influencing factors for the spatial pattern of COVID-19 may change with time ongoing while this was ignored in current other researches.

Hubei province is located in Central China and its capital is Wuhan city where the first wave of COVID-19 was detected. On 23 January 2020, after lockdown of Wuhan City by the Chinese government to prevent the spread of COVID-19, the airline and train transportation was close off strictly so the outflow of people from Wuhan City to other provinces can only take the road transportation and can only go to the 6 adjacent provinces of Hubei instead of other provinces. Thus, the research of spatiotemporal variability of COVID-19 and the influencing factors in these 6 provinces will have enlightening and reference significance for controlling the spread of the COVID-19 and similar future epidemic.

Therefore, this research proposed an assumption that the COVID-19 diffusion would be related to abstract aspects of flow and access. These two aspects would include multiple specific factors, like the population, road networks, elevation, and some other one. All these factors were categorized to a comprehensive analysis framework comprising three dimensions of socio-economic, geography and transportation (SGT), so as to obtain the COVID-19 diffusion characteristics. Based on such assumption and framework, an according case study was also conducted for the NCCC in the first circle of six provinces around Hubei Province in the first four weeks after COVID-19 outbreak and the lockdown on January 23, 2020. The case study firstly detected the spatiotemporal pattern of the COVID-19 diffusion, especially the spatiotemporal variability between these adjacent 6 provinces. Further correlation between COVID-19 and possible influencing factors from SGT was analyzed, as well as further specific regression equation were explored, to identify the key factors affecting COVID-19 spread and spatiotemporal variability. In addition, changes in factors at different weeks in the early stages were also observed and analyzed to verify the proposed hypothesis in this paper. Finally, related epidemic prevention and control measures for risk management were provided based on such analysis and verified hypothesis in this research.

## Data and methods

### Study area

**Fig 1:**
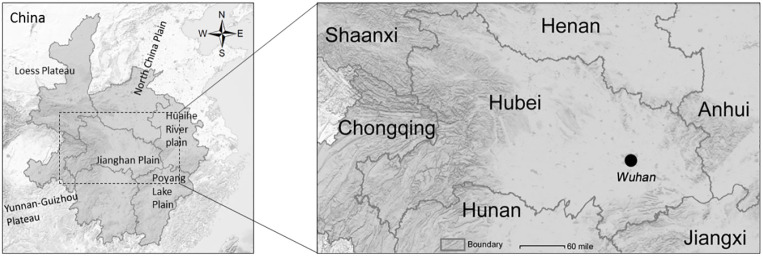
The location of Hubei province and its surrounding six provinces. (figure was drawn by author based on USGS basic information for illustrative purposes only, USGS National Map Viewer (public domain): http://viewer.nationalmap.gov/viewer/).

Hubei Province borders Anhui Province and Jiangxi Province in the East, Hunan Province in the south, Henan Province in the north, Shaanxi Province and Chongqing Municipality in the West. The 6 provinces around Hubei are very important areas with frequent mobility of people flow and dense transportation networks. It is also the most densely populated area in China (Fig 1).

The central part of Hubei Province is Jianghan plain, with flat terrain, dense river network and road network. In the western region, Hubei Province is covered with high mountains, leading to Shaanxi with Loess Plateau in the northwest and Chongqing, next to Yunnan-Guizhou Plateau in the southwest. The regional GDP of Hubei Province in 2019 was 658.4 billion U.S. dollar. According to the bulletin of the seventh national census in 2020, the permanent population of Hubei Province was 57.75 million. While its capital, Wuhan city, located in the east of Jianghan Plain in Hubei Province, has a total area of 8569.15 square kilometers and a permanent resident population of 12.33 million in 2020.

Henan Province, in the north of Hubei, is high in the West but low in the East. It is a part of North China Plain, adjacent to Shaanxi Province in the West and Anhui Province in the East.

Hunan Province, in the south of Hubei, similarly high in the West but low in the East, connected to Yunnan-Guizhou Plateau in the West and Jianghan Plain in the north.

To the west of Hubei Province are Shaanxi Province and Chongqing Municipality. The Loess Plateau in Shaanxi accounts for 40% of the province area. Chongqing is known as "mountain city" due to Mountainous.

Anhui Province and Jiangxi Province are in the east of Hubei. The Huaihe River plain area accounts for 30.48% of Anhui area. Jiangxi is dominated by hills and mountains; Basins and valleys are widely distributed, and Poyang Lake Plain is located in the north.

All these location information is shown in Fig 1 as below.

### SDA-SGT factors base on flow-access frame

In order to fully understand the impact of various factors on the spread of COVID-19, this study set up an abstract frame of flow-access logical relationship for the analysis, and assumes that factors come from three dimensions of socio-economic, geography, and transportation related to flow-access will affect the spread of COVID-19. The logical relationship is as follows:

#### Flow

Firstly, without a flow, the virus source would be difficult to spread [15]. Population is an important flow basis. People always tend to gather in economically developed cities, so GDP is also included in flow category.

#### Access

The flow has to spread with access. The common access selected in this study is the number of main pass and the length of road networks. The number of main pass is the important access from Hubei Province to the surrounding six provinces, including Expressway mainly. The length of road networks is another important access for the people flow and COVID-19 spread within the surrounding 6 provinces. Considering the air transportation was strictly restricted by government after the lockdown of Wuhan and the shipping transportation has very small proportion of passenger traffic (no more than 2.5% since 2001) [28], these two factors were not included in the transportation dimension.

In addition, this research assumes that the terrain also has an impact on the number of main pass and the length of road networks. If the terrain is complex and mountainous, it will bring more difficulties in road construction. Thus, the average elevation is also categorized in access.

Further, the total area, the distance to Wuhan, and the linear length of adjacent boundary between two provinces will also affect the mobility efficiency of people flow. Therefore, area, distance to Wuhan and linear length of adjacent border are also categorized in access.

The size of the factor scale is assumed to affect the spreading scale of the COVID-19. In addition, the spreading efficiency is assumed to relate with density of factors. Therefore, four indicators related to density were selected: density of population, GDP per capital, density of road networks and passenger traffic volume.

All the 12 indicators come from three dimensions: socio-economic, geography and transportation (SGT). Meanwhile, they can also be categorized in another three perspectives: scale, density and access (SDA). The classification of 12 indicators is shown below (Table 1).

**Table 1:** The classification of selected 12 indicators for correlation analysis.

	Socioeconomic	Geography	Transportation
**Scale**	Population(Pop)	Area of province (AP)	Length of road networks(LR)
	Gross Domestic Product(GDP)		
**Density**	Density of population(DP)		Density of road networks(DR)
	GDP per capita(GDPP)		Passenger traffic volume(PV)
**Access**		Distance to Wuhan(DW)	Main pass(MP)
		Length of Adjacent border(LB)	
		Average elevation(AE)	

### Analysis methods

#### Statistical description

**Fig 2:**
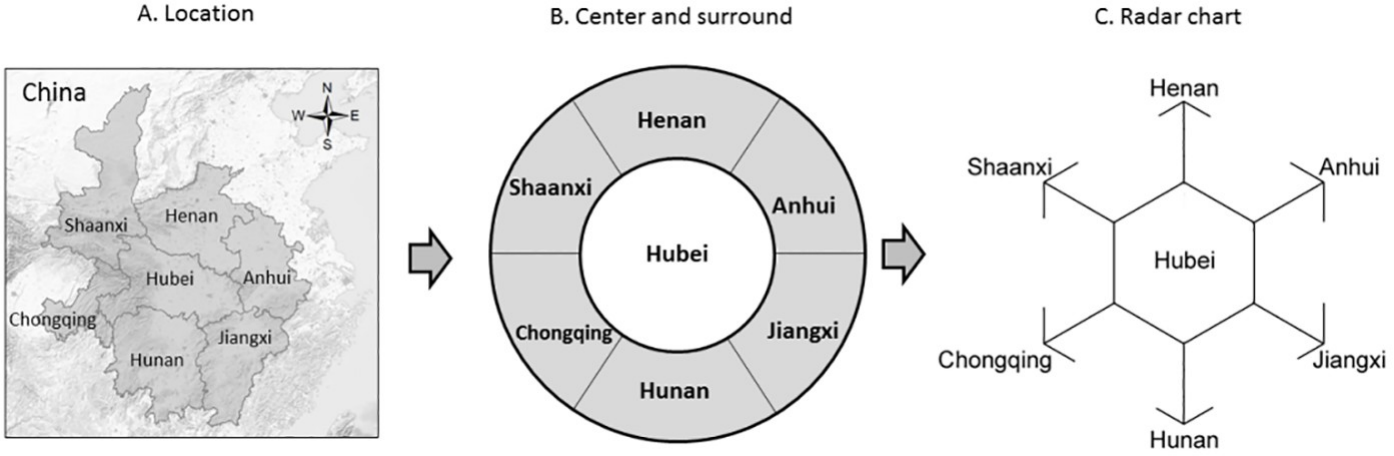
The location of six provinces around Hubei and the corresponding form of radar chart. (figure was drawn by author, A is based on USGS basic information for illustrative purposes only, USGS National Map Viewer (public domain): http://viewer.nationalmap.gov/viewer/).

In the descriptive statistics of 6 provinces for 4 weeks, this study used the method of radar chart. This is mainly because the radar chart is very consistent with the spatial position relationship between the six provinces and Hubei Province (Fig 2). Using the radar chart can more easily and clearly show the spatial variability of the NCCC in six provinces for four weeks, as shown in the figure below (Fig 2).

#### Analysis of variance, correlation and regression

Analysis of variance was used to detect whether there was a significant difference of NCCC in different provinces for 4 weeks.

If there is significant difference, the influencing factors of the difference would be obtained by correlation and regression analysis. The correlation between the NCCC and the selected influencing factors would be analyzed before regression to find the correlation level. Pearson coefficient was selected for correlation analysis.

Multiple regression analysis is carried out based on correlation analysis. After multicollinearity detection, the regression model without multicollinearity is established. For the factors with multicollinearity problem, the univariate regression method is used to establish the regression model.

The general equation for curve fitting by regression model is as follows:

$$Y_{ij}=\beta_{0}+\beta_{1}x_{1}+\beta_{2}x_{2}+\cdots +\beta_{n}\beta_{n}$$ (1)

Where, *Y*_*i*_ is the NCCC in province *i* for week *j*; *x*_*n*_ is the detected significant factor; *β*_*n*_ is the regression coefficient.

### Data collection and processing

#### Data collection

The statistical data of various factors used in this research are from the statistical year-books of National Bureau of statistics (https://data.stats.gov.cn/), the NCCC is from the website of the National Health Commission of the People’s Republic of China (N. H. C. of the People’s Republic of China, http://www.nhc.gov.cn/). Note that the confirmed cases data of COVID-19 analyzed in this research is cumulative values. Further, in order to avoid the epidemic affecting the accuracy of data, the population, GDP, and passenger volume in the six provinces are all based on the data at the end of 2019.

#### Study period

**Fig 3:**
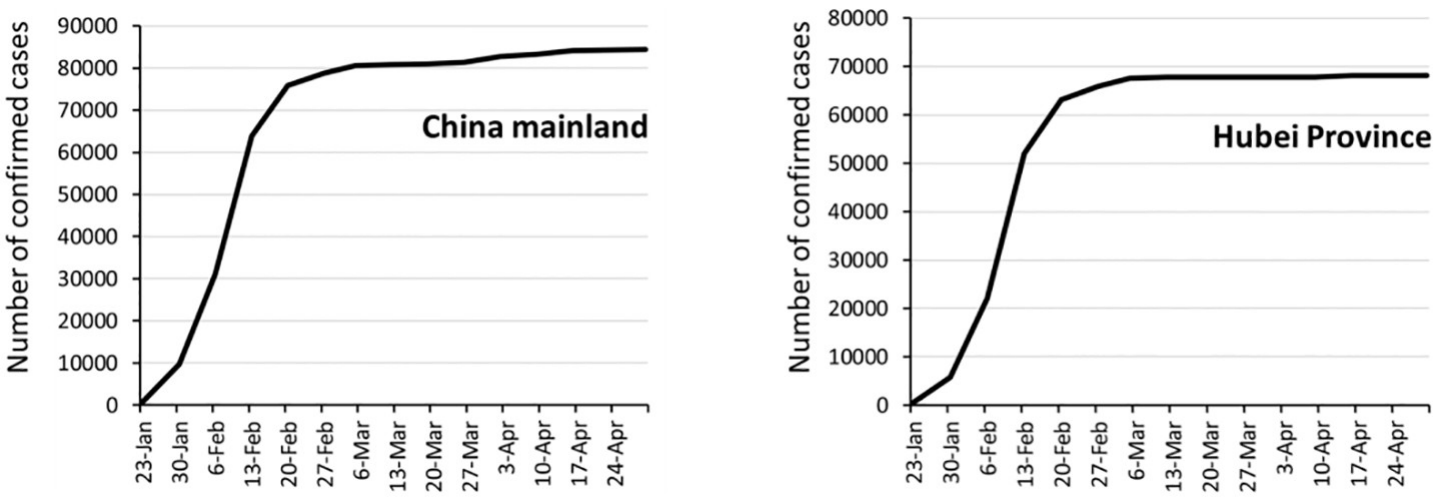
The number change of NCCC in China and Hubei province. (figure was drawn by author).

Within two months after the COVID-19 outbreak and the shutdown of Hubei province and Wuhan city, the number change of confirmed cases in China and Hubei Province is shown in the figure below:

It can be seen from the Fig 3 that, the NCCC mainly increased in the first four weeks since the lockdown of Hubei and Wuhan on January 23 2020, and then gradually stabilized due to the controlling policy and execution. Therefore, the study period selected in this research is that first four weekly nodes from January 23 to February 21. These time nodes are February 1, 7, 14 and 21.

#### Data processing

Excel software was used to sort out the data and do primary analysis, the SPSS software was used for analysis of variance, correlation and regression.

## Results

### Regional difference

**Fig 4:**
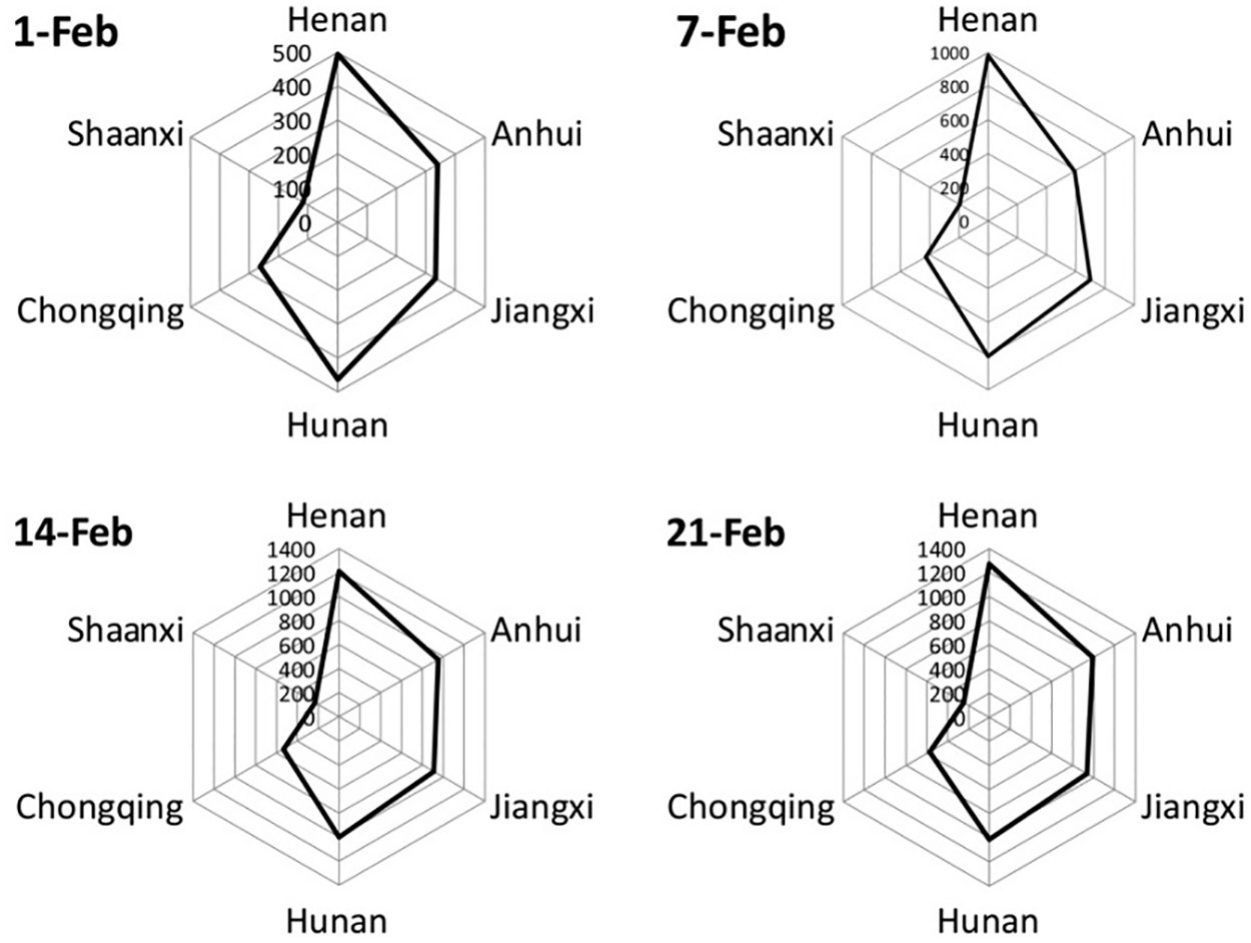
The spatial variability of NCCC of COVID-19 in 6 provinces from February 1 to 21. (figure was drawn by author).

**Fig 5:**
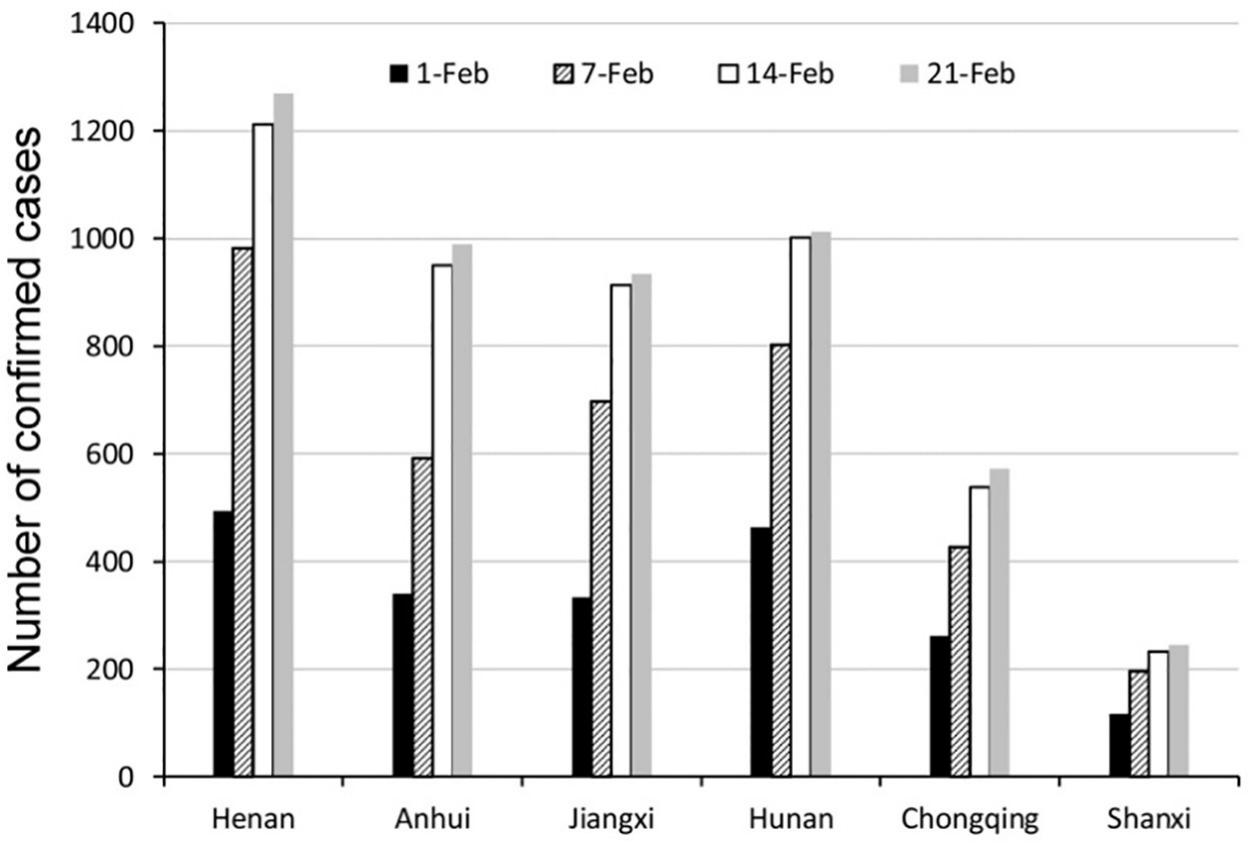
The change of NCCC in 6 provinces from February 1 to 21. (figure was drawn by author).

Within 4 weeks after lockdown of Hubei province on January 23, the NCCC change in 6 provinces around Hubei is shown in Figs 4 and 5 as below.

In the two figures above, it would be more convenient to observe the temporal variability of the NCCC for the six provinces in Fig 5. While Fig 4 could make the observation of the spatial variability of NCCC among the six provinces more clear.

It can be seen in Fig 5 that although the NCCC of six provinces are increasing in four weeks, the growth rate is faster in the first two weeks but slower in the last two weeks. Fig 4 illustrated that there are obvious spatial variability in the NCCC among the six provinces. Generally, the characteristics of regional differences are described as the following (Table 2):

1. For the two western provinces, Shaanxi and Chongqing, the NCCC is less than that in other four provinces along all four weeks. The mean and standard deviation of NCCC in Shaanxi is the lowest (197 ± 58), followed by Chongqing (449± 140).
2. However, for Henan Province adjacent to Shaanxi and Hunan Province adjacent to Chongqing, the NCCC per week is higher than that in other four provinces. Moreover, Henan and Hunan locate in the direction of north-south. Henan has the biggest NCCC with a mean value and standard deviation of 989 ± 353, followed by Hunan (820 ± 257). The strange phenomenon here is that, for the two neighbor provinces of Shaanxi and Henan, the former always has the minimum NCCC, but the latter always has the maximum. These two adjacent provinces have the biggest difference of NCCC (792±295).
3. Anhui and Jiangxi provinces in the East are always has a medium level of NCCC, higher than Chongqing and Shaanxi, but lower than Henan and Hunan. The mean and standard deviation of NCCC in Jiangxi ranked third (720 ± 279), and Anhui ranked fourth (718 ± 309).

**Table 2:** The mean value and standard deviation of NCCC in six provinces for four weeks.

	Henan	Anhui	Jiangxi	Hunan	Chongqing	Shaanxi
**NCCC**	989±353	717.5±309	720±279	820±257	449±140	197±58

In order to explore whether the difference observed in the Fig 4 is significant, further analysis of variance for the NCCC data was performed and the result shows that the significance of variance homogeneity test is *p* = 0.178 > 0.05, indicating that the variance is homogeneous, and analysis of variance is reasonable to use. Consequently, the difference between different provinces is analyzed to be significant (*p* = 0.005 < 0.01), as shown in Table 3.

**Table 3:** The significant differences of NCCC between every two provinces.

	Mean Difference (significance)			
	Henan	Anhui	Jiangxi	Hunan
**Chongqing**	540*(0.008)	-	-	-
**Shaanxi**	792* (0.000)	521 * (0.01)	523* (0.009)	623* (0.003)

In Table 3, it can be seen that there is significant difference between Chongqing and Henan, with mean difference of 540 cases, *p* = 0.008< 0.01. While for Shaanxi, except Chongqing, there are significant differences between it and the other four provinces, of which Henan Province still has the most significant difference, with mean difference of 792 cases, *p* = 0.000. There is no significant difference between other provinces. In the later section of multifactor correlation analysis, this study attempts to explore the potential driving factors of such significant differences of NCCC in six provinces within four weeks.

### Correlation analysis

The results of correlation analysis between the NCCC in four weeks for six provinces and various selected factors are shown in the Table 4.

**Table 4:** Coefficient of Correlation analysis between NCCC and SGT factors.

		1-Feb	7-Feb	14-Feb	21-Feb
**Socioeconomic**	Population	.812*	.833*	0.808	.817*
	Density of population	0.685	0.677	0.701	0.729
	Gross Domestic Product	0.797	0.785	0.764	0.774
	GDP per capita	-0.605	-0.722	-0.733	-0.724
**Geography**	Distance to Wuhan	-0.592	-0.619	-0.712	-0.688
	Length of Adjacent border	0.767	0.721	0.569	0.566
	Area of province	0.101	0.134	0.065	0.039
	Average elevation	-.838*	-.852*	-.943**	-.940**
**Transportation**	Main pass	.912*	.909*	0.802	0.799
	Passenger traffic volume	0.682	0.659	0.523	0.521
	Length of road networks	.895*	.915*	.889*	.888*
	Density of road networks	0.219	0.168	0.201	0.229

*Correlation is significant at the 0.05 level (two-tailed).

**. Correlation is significant at the 0.01 level (2-tailed).

It can be seen from Table 4 that, for the first two weeks, the NCCC is significantly negatively correlated with the average elevation, but significantly positively correlated with the population, the number of main pass and the length of road networks. The highest correlation in the first week is the number of main pass, with coefficient of r = 0.912, *p* = 0.011. The influence in the first week upon the NCCC was ranked as follows: main pass > length of road networks > average elevation > population. The highest correlation in the second week was length of road networks, r = 0. 915, *p* = 0.010. The influence in the second week on the NCCC is ranked as follows: length of road networks > main pass > average elevation > population.

In the third week, the NCCC was significantly positively correlated with the length of road networks but negatively correlated with the average elevation. The correlation coefficient of average elevation was higher than that of length of road networks, and the significant level was *p* < 0.01.

In the fourth week, the NCCC was significantly positively correlated with the population and the length of road networks, but negatively correlated with the average elevation. The correlation coefficient of average elevation was the largest, and the significant level was *p* < 0.01. The influence on the NCCC was ranked as follows: average elevation > length of road networks > population.

Correlation analysis shows the following characteristics:

1. **Comparison among factors**: in four weeks, correlation analysis show that there’s four factors significantly related to the NCCC, including population, average elevation, number of main pass and length of road networks. Among them, the average elevation and the length of road networks are significantly correlated for all 4 weeks, the population is significantly correlated for 3 weeks, and the number of main pass is significantly correlated for the first 2 weeks. In the four weeks, the highest correlation coefficient is the average elevation in the third week (r = -0. 943, *p* = 0.005), and the lowest correlation coefficient is the population in the first week (r = 0. 812, *p* = 0.05).
2. **Comparison between factor categories of SGT**: in the first week, the largest correlation coefficient is the number of main pass. In the second week, the largest correlation coefficient is the length of road networks. In the third and four week, the largest correlation coefficient is the average elevation. The higher the elevation, the more complex the terrain, and the more difficult the road construction. Thus, the number of main pass and the length of road networks are related to geography and transportation. Therefore, transportation and geography are important association dimensions.
3. **Comparison between factor categories SDA**: the population and the length of road networks belong to Scale category, and the average elevation and the number of main pass belong to Access category. Actually, the length of road networks is also the access of domestic circulation for each province. The population provides the flow of virus spreading, while traffic, average elevation, length of road networks and the number of main pass determine the access of virus spreading. Generally, for the factors of average elevation, number of main pass and the length of road networks, all of them related to the Access category. Thus, briefly, the Access plays a key role for COVID-19 spread.

Further, before the regression analysis, a correlation between independent variables was also analyzed to explore the potential internal correlation. The results are shown in the following Table 5 and all the significant correlation coefficient were marked bold.

**Table 5:** Coefficient matrix of Correlation analysis between SGT factors.

	DW	LR	AP	AE	Pop	DP	GDP	GDPP	MP	PV	RL	RD
DW	1	-0.107	-0.428	0.8	-0.411	-0.06	-0.334	0.804	-0.395	-0.151	-0.588	0.355
LR		1	0.267	-0.296	0.699	0.489	0.727	-0.275	.932**	.929**	0.728	0.088
AP			1	0.071	0.307	-0.381	0.27	-0.538	0.317	0.504	0.388	**-.930** **
AE				1	-0.599	-0.581	-0.544	0.684	-0.572	-0.219	-0.708	-0.242
Pop					1	0.76	.986**	-0.692	.855*	.816*	.968**	0.011
DP						1	0.78	-0.268	0.592	0.456	0.659	0.642
GDP							1	-0.577	.840*	.838*	.938**	0.061
GDPP								1	-0.582	-0.418	-0.783	0.402
MP									1	.901*	.909*	0.037
PV										1	0.808	-0.16
RL											1	-0.071
RD												1

*Correlation is significant at the 0.05 level (two-tailed).

**. Correlation is significant at the 0.01 level (2-tailed).

It can be seen from the Table 5 that there are extremely significant correlations between some factors (P < 0.01), such as population and GDP. There were also significant correlations among other factors (P < 0.05), such as GDP and main pass. These significant correlations indicate that there may be multicollinearity, and individual factors need to be eliminated through stepwise regression in the establishment of multiple regression equations.

### Regression analysis

#### Multiple-regression analysis

Correlation analysis showed that the NCCC were significantly affected by demographic, geographical and traffic factors. Based on correlation analysis, the NCCC and the four influencing factors in Table 4 were further analyzed in four time stages to obtain the main driving factors of the spatial variability of NCCC. Because the main influencing factors are different in each week, a stepwise analysis of multiple-regression is used to obtain the equation, the results show that:

In the first week, the average elevation and the number of main pass showed significant correlation with the NCCC, with a significant level of *p* = 0.018 < 0.05. A regression analysis model was established between the NCCC, the average elevation (AE) and the number of main pass (MP). The optimal regression equation was NCCC = 182.121 + 36.406 MP -0.174AE, R^2^_adj_ = 0.967. The standardized regression equation is: NCCC = 0.643 MP -0.470 AE. The F value of ANOVA was 73.289, reaching a very significant level (*p* = 0.003 < 0.01).

In the second week, only the length of road networks showed significant correlation with the NCCC, the other three variables were excluded, indicating that their variables were not independent of each other, and there was multicollinearity in multiple regression. Later, univariate regression analysis will be conducted for various factors in the second week one by one with the NCCC.

In the third week, the average elevation and the length of road networks showed significant correlation with the NCCC, with a significant level of *p* = 0.017 < 0.05. A regression analysis model was established between the NCCC, the average elevation (AE) and the length of road networks (LR). The optimal regression equation was NCCC = 164.795+42.435 LR -0.604AE, R^2^_adj_ = 0.979. The standardized regression equation is: NCCC = 4.829 LR -6.839AE. The F value of ANOVA was 117.270, reaching a very significant level (*p* = 0.001 < 0.01).

In the fourth week, the average elevation and the population showed significant correlation with the NCCC, with a significant level of P = 0.017 < 0.05. A regression analysis model was established between the NCCC, the average elevation (AE) and the population (Pop). The optimal regression equation was NCCC = 830.901+5.977Pop -0.693AE, R^2^_adj_ = 0.972. The standardized regression equation is: NCCC = 4.274Pop -7.582AE. The F value of ANOVA was 89.345, reaching a very significant level (*p* = 0.002 < 0.01).

#### Univariate regression analysis

**Fig 6:**
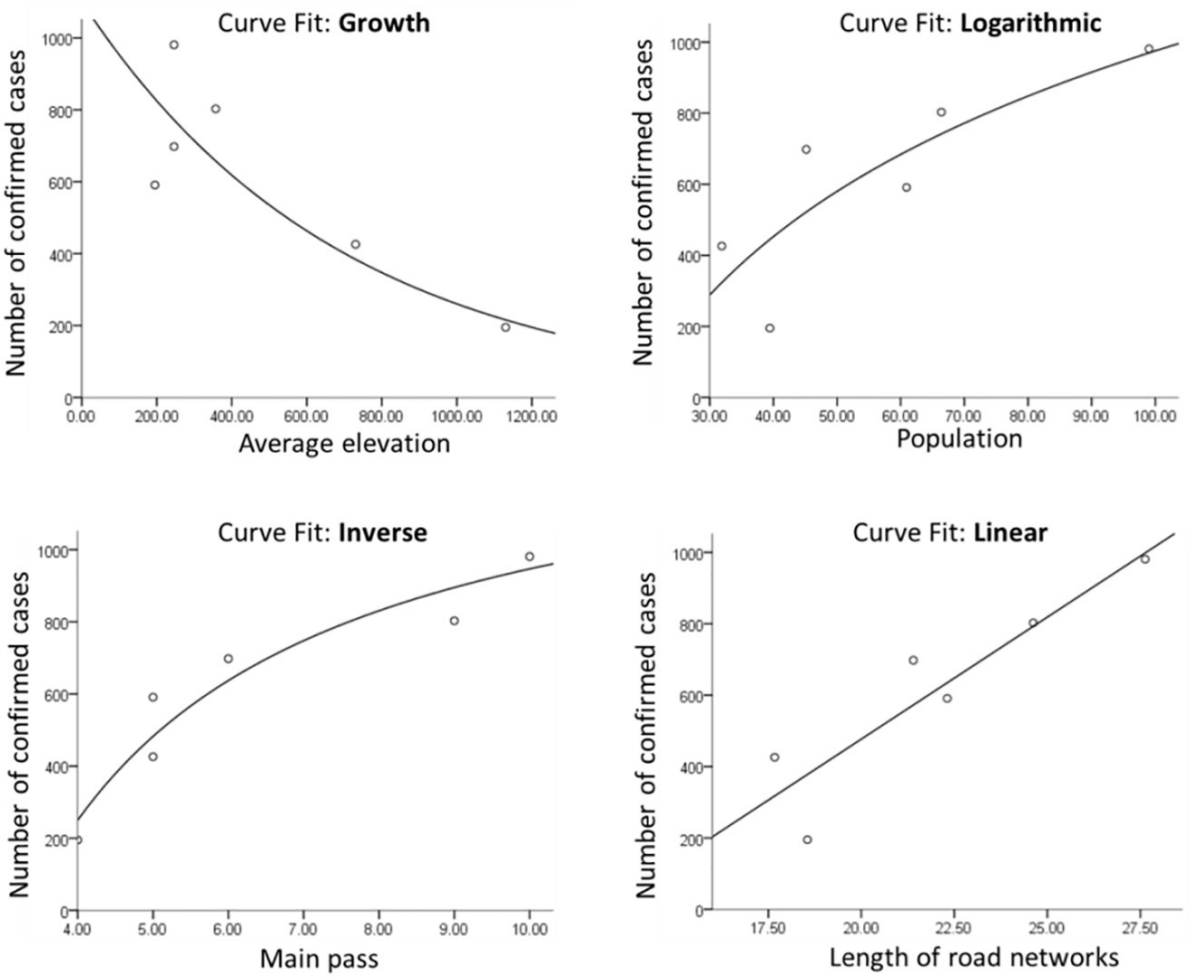
The function relationship between the factors and the confirmed cases in the second week. (figure was drawn by author).

The univariate regression analysis is conducted between the NCCC and various factors in the second week. By the regression analysis, the curve fitting (Fig 6) of NCCC and significant influencing factors in different stages is carried out to obtain the specific functional relationship. The significance test of regression equation found that, when the regression analysis reached the best fitting effect at the level of *p* < 0.05, the regression models of each factor were different. The function relationship with the best fitting effect (significantly highest, *p* minimum) were obtained and the results are shown in the following Table 6 and Fig 6.

**Table 6:** The regression analysis between the factors and the NCCC in the second week.

	Population		Average Elevation		Main pass		Length of road networks	
	R^2^	Prob.	R^2^	Prob.	R^2^	Prob.	R^2^	Prob.
**7-Feb**	0.696	0.039	0.855	0.008	0.919	0.003	0.837	0.01

It can be seen that the function relationship with best fitting effect is the Inverse function between the number of main pass and the NCCC, R square = 0.919, *p* = 0.003. The second is the growth function relationship between the average elevation and the NCCC, R square = 0.855, *p* = 0.008. The worst fitting effect is the logarithmic function relationship between the population and the NCCC, R square = 0.696, *p* = 0.039.

The function results are as follows:

For Average elevation (AE):

$$\text{NCCC}=\text{exp}\;\left(7.003578949291072-0.001441829372325314*\text{AE}\right),\;{\text{R}}^{2}=0.855;$$

For population (Pop):

$$\text{NCCC}=-1651.84825035294+570.5049882130787*\text{log}\;\left(\text{Pop}\right),{\text{R}}^{2}=0.696;$$

For main pass (MP):

$$\text{NCCC}=1411.340887923005-4645.017075442408/\text{MP},{\text{R}}^{2}=0.919;$$

For Length of road networks (LR):

$$\text{NCCC}=68.37378020596179*\text{LR}-890.6077112706716,{\text{R}}^{2}=0.837.$$

On the basis of research for six provinces around Hubei in the second week, the correlation and regression in national scale was also done to detect it is applicable or not. The main pass factor was excluded firstly because not every province borders Hubei. Following correlation analysis showed that the other three factors were significantly correlated with the number of the confirmed cases (*p* < 0.05), especially population (*p* < 0.01). The regression results show that the regression equation of average elevation and population is significant (*p* < 0.01), but the function of best curve fit is different from that of the six provinces around Hubei (Table 7).

**Table 7:** The national correlation and regression analysis in the second week.

		Main pass	Average Elevation	Population	Length of road networks
**NCCC**	Correlation coefficient (Prob.)	——	-.430*(0.018)	.724**(0.000)	.384*(0.036)
	Regression R^2^ (Prob.)	——	0.566(0.000)	0.683(0.000)	0.201(0.114)
	Fit function	——	Growth	S	Cubic

## Discussion

The COVID-19 diffuses with the air. Its diffusion characteristics and related prevention or control is very complex. This situation has put whole world in difficult position for a long time, doing much harm to human health and worldwide social-economy. For a better understand of the COVID-19 diffusion characteristics and related impact factors, this study set up an assumption that the COVID-19 diffusion would be related to abstract aspects of flow and access. Further, this research set up a comprehensive analysis framework of SDA (or SGT) based on such assumption to obtain the diffusion characteristics. An according case study was also conducted with the NCCC in the first circle of six provinces around Hubei for the first four weeks after COVID-19 outbreak and the lockdown. The results show that, the COVID-19 diffusion is indeed related to flow and access, which verifies the hypothesis of this paper. In the following discussion, firstly, this paper outlined the relationship between three dimension of SGT in the analysis framework and COVID-19 diffusion. And then, all the impact factors were summarizes in the two abstract aspects of flow and access.

### Spatiotemporal variability

Many researches detected statistically significant spatial clusters of COVID-19 at various levels in the early stage of COVID-19 outbreak [16–18]. Xie and Wang found that at city level there’s ‘high-high’ clustering type in central China around Wuhan city [17, 18]. The evaluation results by Kang using geographical and distance-based neighbourhoods showed that COVID-19 is highly likely to spread between geographically adjacent regions. While our research found that, although there is overall spatial aggregation in central China, there’s significant differences among the provinces around Hubei. Exactly, Chongqing-Shaanxi in the West of Hubei performed significant differences with other 4 provinces in the East (*p* < 0.05). Especially for Shaanxi, it has the greatest difference with its adjacent Henan Province, with mean difference of 792 cases, *p* = 0.000. This overall spatial pattern is similar to the result in Xie [18], but the strange phenomenon and interesting result in this research is that why provinces spatially adjoin, but NCCC has the biggest difference? Further, Henan and Hunan province in the direction of North-South has bigger NCCC than that of the provinces in direction of East-west, but such difference in direction is not significant (*p* > 0.05).

What factors lead to this adjacent but most different performance?

Current studies have pointed out that population, temperature, GDP and other factors have affected the spread of Covid-19 [15, 19, 25]. While in this research, the correlation analysis showed that the population, average elevation, number of main pass and the length of road networks were significantly correlated with the NCCC.

#### Spatial variability

Xiong concluded that the mean elevation has no association with Covid-19 at the prefecture level in Hubei province from 23 January to 18 February 2020. But at the county level from 26 January to 18 February 2020 the mean elevation are extremely significantly correlated with Covid-19 [19], and this conclusion is similar to this research. Results here indicate that the highest correlation coefficient belongs to the average elevation (r = 0. 943, *p* = 0.005), and this factor was significantly correlated in all four weeks. It means that the complexity of the terrain effectively prevents the spread of the COVID-19 in the province domestic. Moreover, complex terrain also affects another significant factor in this study, the length of road networks. The length of road networks is also significantly correlated along all four weeks. The complex terrain brings great difficulty to the construction of the roads, also affects the length of road networks, finally affecting the spreading of the COVID-19 and the NCCC.

Among the six provinces, Shaanxi has the highest average elevation of 1130m, meanwhile the smallest NCCC (197 ± 58). Chongqing has the second high level of average elevation, while with NCCC ranked second from bottom. Both of the average elevation of Henan Province and Hunan Province is 246m, ranking second from bottom among the six provinces, and their NCCC also ranks first and second high. Although Shaanxi is adjacent to Henan and Chongqing is adjacent to Hunan, the huge difference of average elevation between these adjacent provinces leads to the strange phenomenon of the largest spatial difference for NCCC.

#### Temporal variability

Some research have detected the spatial variability and aggregation of COVID-19, and others also observed the changes in time, but no study notice the influencing factors may change over time [16, 19, 29]. Although Xiong found that the influencing factors were different at two levels, but he did not analyze them in different time periods [19]. Our research found that the significant correlation factors were changing in the first four weeks after lockdown of Wuhan city.

Specifically, the largest correlation coefficient in the first week belongs to the number of main pass (r = 0.912), and in the second week it belongs to the length of road networks (r = 0.915), both of which are traffic related factors, and the correlation coefficient is greater than that of population and average elevation. The highest correlation coefficient in the third and fourth weeks belongs to the average elevation. This change with time going shows that there is significant temporal variability in relevant factors. The reason may be that in the first week after the lockdown of Wuhan city and Hubei province on January 23, the number of main pass is still an important reason for the NCCC increase in each province. With the time going, the lockdown began to play an important role in preventing the spread of COVID-19 between provinces, and the number of main pass began to lose its influence on COVID-19. It is no longer the reason for the NCCC increase in each province. Thus, in the second week, the traffic factor of the length of road networks started to play the most important role, which provides the access of population flow mobility. In the third and the fourth week, with the restriction of mobility among cities in all provinces, the complex terrain factors of average elevation began to play the most important role, preventing the flow range of people and finally limiting the spread of the COVID-19.

### Flow-access

#### Population and flow

The population size provides the potential source, scale and flow of COVID-19 inflection. Without people, the COVID-19 is difficult to spread extensively. The bigger the population size, the larger scale and range the COVID-19 spread. Indeed, many studies pointed out that the population is important factor driving the COVID-19 although their findings were specifically different [15, 19, 22]. Jia and Xie captured population movements were the main factors affecting the epidemic spread [15, 18]. Jahangiri, Sannigrahi and Xiong concluded that total population is significant factor association with COVID-19 [19, 24, 25]. Xiong and Wong also shows that population density has significant correlation with cumulative infection cases in both in Hubei province, China and the U.S. at the county level [19, 30].

Howerver, in this research, analysis results show that population size is a significant factor affecting the NCCC, but population density is tested not a significant factor, which is similar with the results by Xiong at the prefecture level [19]. That means although the people gathers, resulting in a big population density, without a big population size, the NCCC cannot be increased. Population size provides the basis for people flow and potential COVID-19 flow, while roads and terrain provide access to these two flows.

#### Transportation, geography and access

Few studies have noticed the impact of traffic on the epidemic spread. Xie uses the ‘Distance from Wuhan (km)’ factor to describe aspect of ‘Traffic accessibility’. Xie concluded that traffic accessibility has influence on the epidemic spread at city level [18]. While, our study found that the factor of ‘Distance from Wuhan’ has no significant relationship at the province level. The significant influence factor about transportation we found is the length of road networks and the number of main pass, which is ignored by other research. In addition, the correlation coefficient from the length of road networks and the number of main pass is always greater than that of the population, and both of them belong to transportation factors. It can be seen that transportation plays the most important role in this study.

#### Transportation

From the transportation factor data, Shaanxi does has the least number of main pass (only 4) connected with Hubei among the six provinces, and the length of road networks ranked second to bottom (185,500km), only longer than the last province Chongqing (176,700 km). While both Shaanxi and Chongqing is in the west of Hubei Province.

Henan Province, which is adjacent to Shaanxi, has the largest number of main pass connected to Hubei (10), followed by Hunan (9). In terms of the length of road networks, Henan also has the largest total length (276,300 km), followed by Hunan, ranking second (246,200 km). While both Henan and Hunan is located in the north-south direction of Hubei Province.

Why is there the least number of main pass and total length of road networks in the west, but the biggest in the north-south direction?

#### Geography

It can be seen from the location map that the terrain of Hubei Province is great plain on both sides of the north and south, with few high mountains, and has the widest straight-line border length in the north-south direction, of which the longest is 460km with Henan, followed by 456km with Hunan (Fig 1). These factors result in Henan and Hunan having the largest number of main pass.

In the West and northwest, there are many high mountains in Hubei Province, which are not conducive to the construction of roads. Especially, Shaanxi has the shortest straight-line border length (215km) with Hubei, which leads to the least number of main pass in Shaanxi.

Within the six provinces, Henan has the largest total length of road networks and Hunan ranks second. At the same time, the average elevation of the two provinces ranks the second from the bottom, which shows that both provinces are great plain. The leveling of terrain reduces the difficulty of building roads, so geographical factors and traffic factors are closely related.

#### Access

To sum up, no matter the geographical factor of average elevation, or the number of main pass and the total the length of road networks, these factors determine the flow access of people flow and COVID-19 flow. If there is only flow without access, the COVID-19 would be difficult to spread out of a region. In this study, the average elevation associated with the main pass and the total length of road networks become the determinants of access for COVID-19 flow spread.

#### Other factors

Xiong found that the NCCC had a positive and extremely significant correlation GDP at the prefecture level in Hubei province and negatively associated with land area at the county level [19]. But in our study result, both GDP and land area has no significant correlation with NCCC in the 6 provinces around Hubei.

## Conclusion

The COVID-19 diffuses with the air. Its diffusion characteristics and related prevention or control is very complex. This study proposed an assumption that the COVID-19 diffusion would be related to flow and access, and set up a comprehensive analysis framework of SDA (or SGT) based on such assumption to obtain the diffusion characteristics. An according case study was also conducted based on the NCCC in the first circle of six provinces around Hubei for the first four weeks after COVID-19 outbreak and the lockdown. The results show that, the COVID-19 diffusion is indeed related to flow and access, which verifies the hypothesis of this paper. Thus, the hypothesis, analysis framework and corresponding cases study proposed in this paper would highlight a direction for analysis and corresponding prevention of similar air-based virus diffusion.

In this paper, there’s only one case study. For better supporting of the hypothesis and analysis framework proposed in this paper, more case study, discussion and verifications are necessary. Meanwhile, in this specific case study, considering the regional limitation and the time period limitation, the case analysis results, especially the regression equation and the included specific parameters, would have certain particularity and applicable limitation for generality. That means, when applied to the analysis of other regions and other periods, the correlation coefficient and regression equation may be different.

Basically, in this research case, the general relationship and specific characteristics of COVID-19 diffusion of the NCCC in the first circle of six provinces around Hubei for the first four weeks after COVID-19 outbreak and lockdown are the following:

1. Analysis of variance showed that there were significant spatial differences in the NCCC. Shaanxi and Chongqing in the West were less than the other four provinces, ranking the last and second respectively. Particularly, Shaanxi in the northwest is significantly different from the four provinces in the East, and the difference with the adjacent Henan is the largest (792 cases).
2. Correlation analysis showed that the average elevation and the length of road networks were significantly correlated in all four weeks, the number of main pass is significantly correlated in the first two weeks, and the population is significantly correlated except the third week. In the four weeks, the highest correlation coefficient belongs to the average elevation in the third week (r = 0.943, *p* = 0.005).
3. For temporal variability, the regression analysis showed that in the first week, the average elevation and the number of main pass showed significant correlation with the NCCC. The optimal regression equation was NCCC = 182.121 + 36.406 MP -0.174AE, R^2^_adj_ = 0.967. The standardized regression equation is: NCCC = 0.643 MP -0.470 AE. In the second week, there’s no significant multiple regression. The result of univariate regression analysis show that the number of main passes has inverse function relationship, the average elevation has growth function, the population has logarithmic function, and the length of road networks has a linear correlation. In the third week, the average elevation and the length of road networks showed significant correlation with the NCCC. The optimal regression equation was NCCC = 164.795+42.435 LR -0.604AE, R^2^_adj_ = 0.979. The standardized regression equation is: NCCC = 4.829 LR -6.839AE. In the fourth week, the average elevation and the population showed significant correlation with the NCCC. The optimal regression equation was NCCC = 830.901+5.977Pop -0.693AE, R^2^_adj_ = 0.972. The standardized regression equation is: NCCC = 4.274Pop -7.582AE.
